# Rehabilitation Exercise and psycholoGical support After covid-19 InfectioN’ (REGAIN): a structured summary of a study protocol for a randomised controlled trial

**DOI:** 10.1186/s13063-020-04978-9

**Published:** 2021-01-06

**Authors:** Gordon McGregor, Harbinder Sandhu, Julie Bruce, Bartholomew Sheehan, David McWilliams, Joyce Yeung, Christina Jones, Beatriz Lara, Jessica Smith, Chen Ji, Elaine Fairbrother, Stuart Ennis, Peter Heine, Sharisse Alleyne, Jonathan Guck, Emma Padfield, Rachel Potter, James Mason, Ranjit Lall, Kate Seers, Martin Underwood

**Affiliations:** 1 Department of Cardiopulmonary Rehabilitation, Centre for Exercise & Health, University Hospitals Coventry & Warwickshire NHS Trust, Coventry, UK; 2 Warwick Clinical Trials Unit, Warwick Medical School, University of Warwick, Coventry, UK; 3 Centre for Sport, Exercise & Life Sciences, Coventry University, Coventry, UK; 4 Coventry & Warwickshire Partnership Trust, Coventry, UK; 5 Centre for Care Excellence, University Hospitals Coventry & Warwickshire NHS Trust, Coventry, UK; 6 Department of Anaesthesia and Critical Care, University Hospitals Birmingham NHS Foundation Trust, Birmingham, UK; 7 ICUSteps, Kemp House, London, UK; 8 Department of Respiratory Medicine, University Hospitals Coventry & Warwickshire NHS Trust, Coventry, UK; 9 Patient & Public Involvement Representative, Coventry, UK; 10 Centre for Health Economics at Warwick, Warwick Medical School, University of Warwick, Coventry, UK; 11 Warwick Research in Nursing, Warwick Medical School, University of Warwick, Coventry, UK

**Keywords:** COVID-19, randomised controlled trial, protocol, rehabilitation, exercise, psychological support, mental health, physical health, online

## Abstract

**Objectives:**

The primary objective is to determine which of two interventions: 1) an eight week, online, home-based, supervised, group rehabilitation programme (REGAIN); or 2) a single online session of advice (best-practice usual care); is the most clinically and cost-effective treatment for people with ongoing COVID-19 sequelae more than three months after hospital discharge.

**Trial design:**

Multi-centre, 2-arm (1:1 ratio) parallel group, randomised controlled trial with embedded process evaluation and health economic evaluation.

**Participants:**

Adults with ongoing COVID-19 sequelae more than three months after hospital discharge

*Inclusion criteria:* 1) Adults ≥18 years; 2) ≥ 3 months after any hospital discharge related to COVID-19 infection, regardless of need for critical care or ventilatory support; 3) substantial (as defined by the participant) COVID-19 related physical and/or mental health problems; 4) access to, and able/supported to use email and internet audio/video; 4) able to provide informed consent; 5) able to understand spoken and written English, Bengali, Gujarati, Urdu, Punjabi or Mandarin, themselves or supported by family/friends.

*Exclusion criteria:* 1) exercise contraindicated; 2) severe mental health problems preventing engagement; 3) previous randomisation in the present study; 4) already engaged in, or planning to engage in an alternative NHS rehabilitation programme in the next 12 weeks; 5) a member of the same household previously randomised in the present study.

**Intervention and comparator:**

*Intervention 1:* The Rehabilitation Exercise and psycholoGical support After covid-19 InfectioN (REGAIN) programme: an eight week, online, home-based, supervised, group rehabilitation programme.

*Intervention 2:* A thirty-minute, on-line, one-to-one consultation with a REGAIN practitioner (best-practice usual care).

**Main outcomes:**

The primary outcome is health-related quality of life (HRQoL) – PROMIS® 29+2 Profile v2.1 (PROPr) – measured at three months post-randomisation.

Secondary outcomes include dyspnoea, cognitive function, health utility, physical activity participation, post-traumatic stress disorder (PTSD) symptom severity, depressive and anxiety symptoms, work status, health and social care resource use, death - measured at three, six and 12 months post-randomisation.

**Randomisation:**

Participants will be randomised to best practice usual care or the REGAIN programme on a 1:1.03 basis using a computer-generated randomisation sequence, performed by minimisation and stratified by age, level of hospital care, and case level mental health symptomatology.

Once consent and baseline questionnaires have been completed by the participant online at home, randomisation will be performed automatically by a bespoke web-based system.

**Blinding (masking):**

To ensure allocation concealment from both participant and REGAIN practitioner at baseline, randomisation will be performed only after the baseline questionnaires have been completed online at home by the participant. After randomisation has been performed, participants and REGAIN practitioners cannot be blind to group allocation. Follow-up outcome assessments will be completed by participants online at home.

**Numbers to be randomised (sample size):**

A total of 535 participants will be randomised: 263 to the best-practice usual care arm, and 272 participants to the REGAIN programme arm.

**Trial Status:**

Current protocol: Version 3.0 (27th October 2020) Recruitment will begin in December 2020 and is anticipated to complete by September 2021.

**Trial registration:**

ISRCTN:11466448, 23rd November 2020

**Full protocol:**

The full protocol Version 3.0 (27th October 2020) is attached as an additional file, accessible from the Trials website (Additional file 1). In the interests of expediting dissemination of this material, the familiar formatting has been eliminated; this Letter serves as a summary of the key elements of the full protocol.

The study protocol has been reported in accordance with the Standard Protocol Items: Recommendations for Clinical Interventional Trials (SPIRIT) guidelines.

## Supplementary Information

**Additional file 1.**

## Data Availability

The chief investigator and co-investigators will have access to the final trial dataset. The trial will be reported in accordance with the Consolidated Standards of Reporting Trials (CONSORT) guidelines. Trial results will be published in peer-reviewed open access journals. Requests for data sharing will be reviewed on an individual basis by the chief investigator in consultation with the trial management group and trial steering committee, after publication of the final trial results. The study will comply with the good practice principles for sharing individual participant data from publicly funded clinical trials and data sharing will be undertaken in accordance with the required regulatory requirements.

