# Correction to: Rehabilitation Exercise and psycholoGical support After covid-19 InfectioN’ (REGAIN): a structured summary of a study protocol for a randomised controlled trial

**DOI:** 10.1186/s13063-021-05045-7

**Published:** 2021-01-26

**Authors:** Gordon McGregor, Harbinder Sandhu, Julie Bruce, Bartholomew Sheehan, David McWilliams, Joyce Yeung, Christina Jones, Beatriz Lara, Jessica Smith, Chen Ji, Elaine Fairbrother, Stuart Ennis, Peter Heine, Sharisse Alleyne, Jonathan Guck, Emma Padfield, Rachel Potter, James Mason, Ranjit Lall, Kate Seers, Martin Underwood

**Affiliations:** 1 Department of Cardiopulmonary Rehabilitation, Centre for Exercise & Health, University Hospitals Coventry & Warwickshire NHS Trust, Coventry, UK; 2 Warwick Clinical Trials Unit, Warwick Medical School, University of Warwick, Coventry, UK; 3 Centre for Sport, Exercise & Life Sciences, Coventry University, Coventry, UK; 4 Coventry & Warwickshire Partnership Trust, Coventry, UK; 5 Centre for Care Excellence, University Hospitals Coventry & Warwickshire, NHS Trust, Coventry, UK; 6 Department of Anaesthesia and Critical Care, University Hospitals Birmingham NHS Foundation Trust, Birmingham, UK; 7 ICUSteps, Kemp House, London, UK; 8 Department of Respiratory Medicine, University Hospitals Coventry & Warwickshire NHS Trust, Coventry, UK; 9 Patient & Public Involvement Representative, Coventry, UK; 10 Centre for Health Economics at Warwick, Warwick Medical School, University of Warwick, Coventry, UK; 11 Warwick Research in Nursing, Warwick Medical School, University of Warwick, Coventry, UK

**Correction to: Trials 22, 8 (2021)**

**https://doi.org/10.1186/s13063-020-04978-9**

Following publication of the original article [1], we were notified that the additional file did not correctly provide a link directly to the full trial protocol.

The original paper has been corrected.

## References

[1] McGregor G (2021). Rehabilitation Exercise and psycholoGical support After covid-19 InfectioN’ (REGAIN): a structured summary of a study protocol for a randomised controlled trial. Trials.

