# Supplementary material for: Rehabilitation Exercise and psycholoGical support After covid-19 InfectioN’ (REGAIN): a structured summary of a study protocol for a randomised controlled trial
Source: Trials. 2021 Jan 6;22:8. doi: 10.1186/s13063-020-04978-9 (PMC7785779; doi:10.1186/s13063-020-04978-9)
Supplement: Supplementary file 1 — Additional file 1. [file 13063_2020_4978_MOESM1_ESM.pdf]

NHS
Health Research Authority

East of England - Cambridge South Research Ethics Committee

The Old Chapel
Royal Standard Place
Nottingham
NG1 6FS

Please note: This is the favourable opinion of the REC only and does not allow the amendment to be implemented at NHS sites in England until the outcome of the HRA assessment has been confirmed.

08 December 2020

Ms Sharisse Alleyne
Warwick Clinical Trials Unit, Warwick Medical School
University of Warwick
Coventry
CV4 7AL

Dear Ms Alleyne,

**Study title:**	**Rehabilitation Exercise and psycholoGical support After covid-19 InfectioN' (REGAIN): a multi-centre randomised controlled trial**
**REC reference:**	**20/EE/0235**
**Amendment number:**	**Substantial Amendment 1**
**Amendment date:**	**06 November 2020**
**IRAS project ID:**	**288362**

The above amendment was reviewed on 16 November 2020 by the Sub-Committee in correspondence.

Ethical opinion

The members of the Committee taking part in the review gave a favourable ethical opinion of the amendment on the basis described in the notice of amendment form and supporting documentation.

The sub-Committee had requested that the following wording is revised in the consent form for the participant interview to appear less coercive:

“In order to take part in a REGAIN interview, you must be happy to answer all of the items on this consent form as Yes. If your answer to an item is No, that is not a problem and you can still submit the consent form, however you will not be able to continue and take part in an interview.”

The applicants updated the Participant Interview Consent Form and had elected to remove the wording to avoid any risk of perceived coercion.

The sub-Committee reviewed the response and was satisfied with the changes.

Approved documents

The documents reviewed and approved at the meeting were:

Document	*Version*	*Date*
Completed Amendment Tool [REGAIN SA1 Amendment Tool]	N/A	06 November 2020
Covering letter on headed paper [REGAIN Cover Letter SA01]	N/A	06 November 2020
Other [REGAIN Participant Follow-up CLMHD Letter V1.0 26Oct2020]	V1.0	26 October 2020
Other [REGAIN Participant Workbook Letter V1.0 26Oct2020]	V1.0	26 October 2020
Other [REGAIN Notifications Wording - Patients V1.0 28Oct 2020]	V1.0	28 October 2020
Other [REGAIN Summary of Changes_ Amendment 1]	N/A	28 October 2020
Other [Response to Committee Query]		04 December 2020
Participant consent form [REGAIN Consent Form V3.0 29Oct2020 - Clean]	V3.0	29 October 2020
Participant consent form [REGAIN Consent Form V3.0 29Oct2020 - Tracked Changes]	V3.0	29 October 2020
Participant consent form [REGAIN Consent Form Participant Interview V3.0 04Dec2020]	3.0	04 December 2020
Participant consent form [REGAIN Consent Form Participant Interview V3.0 04Dec2020 TC.]	3.0	04 December 2020
Participant information sheet (PIS) [REGAIN PIS V3.0 29Oct2020 - Clean]	V3.0	29 October 2020
Participant information sheet (PIS) [REGAIN PIS V3.0 29Oct2020 - Tracked Changes]	V3.0	29 October 2020
Participant information sheet (PIS) [REGAIN PIS Participant Interview V3.0 27Oct2020 Clean]	V3.0	27 October 2020
Participant information sheet (PIS) [REGAIN PIS Participant Interview V3.0 27Oct2020 Tracked Changes]	V3.0	27 October 2020
Participant information sheet (PIS) [REGAIN PIS practitioner interview V3.0 29Oct2020 Clean]	V3.0	29 October 2020
Participant information sheet (PIS) [REGAIN PIS practitioner interview V3.0 29Oct2020 Tracked Changes]	V3.0	29 October 2020
Research protocol or project proposal [REGAIN Protocol v3.0 20201029 - Clean]	V3.0	29 October 2020
Research protocol or project proposal [REGAIN Protocol v3.0 20201029 - Track Changed]	V3.0	29 October 2020

Membership of the Committee

The members of the Committee who took part in the review are listed on the attached sheet.

Working with NHS Care Organisations

Sponsors should ensure that they notify the R&D office for the relevant NHS care organisation of this amendment in line with the terms detailed in the categorisation email issued by the lead nation for the study.

Amendments related to COVID-19

We will update your research summary for the above study on the research summaries section of our website. During this public health emergency, it is vital that everyone can promptly identify all relevant research related to COVID-19 that is taking place globally. If you have not already done so, please register your study on a public registry as soon as possible and provide the HRA with the registration detail, which will be posted alongside other information relating to your project.

Statement of compliance

The Committee is constituted in accordance with the Governance Arrangements for Research Ethics Committees and complies fully with the Standard Operating Procedures for Research Ethics Committees in the UK.

HRA Learning

We are pleased to welcome researchers and research staff to our HRA Learning Events and online learning opportunities– see details at: https://www.hra.nhs.uk/planning-and-improving-research/learning/

**IRAS Project ID - 288362:**	**Please quote this number on all correspondence**

Yours sincerely,

pp

Dr Leslie Gelling
Chair

E-mail: cambridgesouth.rec@hra.nhs.uk

Enclosures: List of names and professions of members who took part in the review

Copy to: Dr Gordon McGregor, University Hospitals Coventry and Warwickshire NHS Trust

East of England - Cambridge South Research Ethics Committee

Attendance at Sub-Committee of the REC meeting on 16 November 2020

Committee Members:

Name	*Profession*	*Present*
Dr Joanne Doleman	Research Governance Manager	Yes
Dr Leslie Gelling	Principal Academic in Adult Nursing	Yes (Chair)

Also in attendance:

Name	*Position (or reason for attending)*
Miss Rebecca Morledge	Approvals Officer
Chelsea Phillips	Approvals Administrator
